# Bone Metabolism Disorder in Epileptic Children

**Published:** 2018

**Authors:** MAaryam NAKHAEE MOGHADAM, Alireza TEIMOURI, Ali KHAJEH, Seyed BAHARE HOSEINI

**Affiliations:** 1 Children & Adolescent Health Research Center, Resistant Tuberculosis Institute, School of Medicine, Zahedan University of Medical Sciences, Zahedan, Iran.

**Keywords:** Anti-epileptic drugs, Bone metabolism, Epilepsy, Children

## Abstract

**Objective:**

There are frequent anti-epileptic drugs used in management of epilepsy. Anti-epileptic drugs may have some complications on bone and vitamin D metabolism. This study aimed to comparison the bone metabolism disorder in epileptic children with healthy child in Zahedan, eastern Iran from Jul 2014 to Jun 2015.

**Materials & Methods:**

This case-control study was performed on bone metabolism disorder in epileptic children between 2014-2015. Forty epileptic children were enrolled based on accessibility scheme and 40 participants randomly selected for control group from those referred to the pediatric ward and clinic of Ali Ebn Abi Talib Hospital and Ali Asghar Clinic in Zahedan City, Sistan & Baluchestan Province, eastern Iran. Blood samples were collected from all participants to assess serum calcium, phosphorus, parathyroid hormone, magnesium, vitamin D, serum albumin, creatinine random urine.

**Results:**

Of 40 epileptic children, 23 (57.5%) and 17 (42.5%) were male and female respectively, The prevalence of lower mean vitamin D levels was 37.5% for patients compared to 12.5% for controls (chi-square=6.667 and *P*=0.010). Of 80 participants, 15 individuals had abnormal PTH levels detected of 2 and 13 for patients and control groups, respectively (chi-square =9.928 and *P*=0.002). Serum calcium and magnesium levels were comparable in both groups. The status of the parameters in the classification of normal and abnormal assessed based on number of medications intake resulted that number of medications intake had no effect on the status of the parameter.

**Conclusion:**

The frequency of hyperparathyroidism and vitamin D deficiency is increased in epileptic children.

## Introduction

Epilepsy is one of major public health problems affecting nearly 50 million people worldwide. It is one of the most common and long-term neurological disorders with a prevalence of 4-10/1000 in developed countries. The prevalence is much higher in children and aged population (1-3). Epilepsy is defined more than two days epileptic seizure without any specific causes more than 24 h. 

Patients with epilepsy are required long-term treatments and estimated that 1% of populations are taking medicine for epilepsy. Due to the long duration of treatment, these patients are at risk of side effects. Metabolic bone diseases and increased risk of fracture are the most important side effects for these patients. The relationship between (AEDs) and the incidence of rickets, osteomalacia and increased fracture is reported (4, 5). The above-mentioned clinical or subclinical abnormalities are observed in 50% or more patients treated with antiepileptic drugs (6). Bone disorders due to antiepileptic drugs are affected by both duration and dose of these medicines and mostly are observed bone disorders with all antiepileptic medications except gabapentin for instance (7). (AEDs) are along with osteoporosis, other bone metabolism disorders and mineral materials such as hypocalcemia (8), hypophosphatemia (9), a decrease in the serum levels of vitamin D (10) and secondary hyperparathyroidism (11, 12). These biochemical changes are causes of being at decreasing bone density risk, osteoporosis and fracture for patients under medication (12). In addition, of mentioned factors along with drugs consumption reducing an amount of activities, sunlight exposure, vitamin D, and calcium have a strong role in bone disorders.

Bone disorders pathogenesis is multifactorial in these patients and including changes in the metabolism of vitamin D, secondary hyperparathyroidism, direct inhibition of intestinal absorption of calcium, changes in the metabolism of vitamin K, reducing in calcitonin, motility, calcium, and exposure to sunlight, and production of endogenous estrogen (13). We studied the relationship between bone metabolic disorders in children with epilepsy who were under long-term treatment and antiepileptic drugs (AEDs).

## Materials & Methods

This case-control study was conducted on 80 children with epilepsy diseases aged from 5 to 15 yr of which 40 were patients and 40 were controls to assess bone metabolism disorders. Sampling was random and easy access from those referred to the Pediatric Ward and clinics of Ali Ebn Abi Talib Hospital and Ali Asghar clinic in Zahedan City, Sistan & Baluchestan Province, Iran from Jul 2014 to Jun 2015. 

The sample size of 80 was estimated based on *P*_1_= 0.6%, *P*_2_=22% and Z _1-α/2_ = 1.96 with the desired precision of 5%, alpha of 0.05 (CI of 95%) and 80% of power. Sampling method for patients and controls was accessible means that referred patients to clinics or admitted to the pediatric ward entered to the case group. Patients with exclusion criteria were excluded from the study. In addition, from those who were not taking antiepileptic drugs, 40 selected for the control group randomly. The control group participants were without any underline diseases and matched with patients based on age and sex. 

Both case and control participants were in the normal range of weight, height, and growth. Blood samples were collected from all participants to assess the serum levels of calcium, phosphorus, PTH, magnesium, vitamin D, albumin and urine calcium and creatinine in random urine sample. Since these paraclinical tests are part of routine checkup for patients with epilepsy every six months, the researcher did not pay cost for but for controls, they did. 

Ethics Committees of Zahedan University of Medical Sciences approved the study for MD thesis coded of 1515. All parents, caregivers, or legal representatives gave informed consent form before participating in the study. 

All statistical analyses were performed using SPSS software, version 13.0 (Chicago, IL, USA). Age, weight, BMI, AED, and levels of calcium were expressed as the mean ± standard deviation (SD) and frequency (percentage). Comparisons of the frequency distribution were conducted by chi-square test. A *P*-value <0.05 was considered statistically significant.

## Results

characteristicsand controls are presented in (Table 1). Between two groups of participants, the frequency of sex distribution was similar (*P*> 0.05). Mean age of participants was 7.95 ± 2.14 with the range of 5-15 years old. Patients had mean age of 8.17± 2.38 years old with maximum age of 13 years old and a minimum age of 5 years old. In healthy subjects, mean age was 7.73±1.88 years old with maximum age of 12 years old and a minimum age of 5 years old. Means of age between healthy people and people with seizure did not show any different. 

The prevalence of low vitamin D was 37.5% in patients compared to 12.5% in controls. (Chi-square=6.667 and *P*=0.010). From 80 participants, 15 individuals had abnormal PHT level distributed of 2(5%) and 13(32.50%) for control and patients participants respectively (chi-square=9.928 and *P*= 0.002) (Table 2). In the cases of Ca and Mg, their levels were similar in both patients and control groups. For P and Ald all participants had normal levels in both case and controls (Table 2). The status of the parameters in the classification of normal and abnormal assessed based on number of medicines intake. In all mentioned parameters, the number of medicines intake had no effect on the status of the parameter of normality in patients (Table 3). 

**Table 1:** Sex distribution of study subjects in case and control

Sex	Control		Case		Total	
	n	%	n	%	n	%
Boys	23	57.5	20	50	43	53.75
Girls	17	42.5	20	50	37	46.25
Total	40	100	40	100	80	100

**Table 2:** The prevalence of vitamin D, calcium, phosphorus, albumin, magnesium hyper parathyroid groups

Variables	Status	Statistics	Participants		x^2^	*P*-value
			Case	Control		
Vitamin D	Abnormal	N	15	5	6.667	0.010
		%	37.50%	12.50%		
	Normal	N	25	35		
		%	62.50%	87.50%		
	Total	N	40	40		
Parathyroid	Abnormal	N	13	2	9.928	0.002
		%	32.50%	5%		
	Normal	N	27	38		
		%	67.50%	95%		
	Total	N	40	40		
Calcium	Abnormal	N	3	1	10.53	0.305
		%	7.50%	2.50%		
	Normal	N	37	39		
		%	92.50%	97.50%		
	Total	N	40	40		
Magnesium	Abnormal	N	1	0	1.13	0.5
		%	2.50%	0		
	Normal	N	39	40		
		%	97.50%	100%		
	Total	N	40	40		
Phosphate	Abnormal	N	0	0	-	-
		%	0	0		
	Normal	N	40	40		
		%	100%	100%		
	Total	N	40	40		
Albumin	Abnormal	N	0	0	-	-
		%		0		
	Normal	N	40	40		
		%	100%	100%		
	Total	N	40	40		

**Table 3:** Chi-square test results to evaluate the effect of the drug on laboratory data only in patients

Varibles	Status	No of medications consumption			Total	Chi- square	*P*-value
		1	2	3			
Vitamin D	Abnormal	4	7	4	15	1.068	0.586
	Normal	10	11	4	25		
	Total	14	18	8	40		
Parathyroid	Abnormal	2	7	4	13	3.569	0.168
	Normal	12	11	4	27		
	Total	14	18	8	40		
Calcium	Abnormal	1	1	1	3	0.389	0.823
	Normal	13	17	7	37		
	Total	14	18	8	40		
Magnesium	Abnormal	1	0	0	1	1.905	0.386
	Normal	13	18	8	39		
	Total	14	18	8	40		
Phosphate	Abnormal	0	0	0	0	-	-
	Normal	14	18	8	40		
	Total	14	18	8	40		
Albumin	Abnormal	0	0	0	0	-	-
	Normal	14	18	8	40		
	Total	14	18	8	40		

## Discussion

Calcium and vitamin D play important roles in bone metabolism and preserving adequate bone mass. This study was performed on 80 children, of which 40 were taking antiepileptic drugs more than 6 months and 40 were healthy. Two groups were matched in age and sex.

Normal status of vitamin D has remarkable influence in children and adolescent who are growing. Our analysis showed that in the level of abnormality, PTH and the level of serum calcium were higher in patients compared to controls. 

Several factors have important role in calcium metabolism and bone disorder such as dark skin, contacts and lack of sufficient additional sunlight and supplementary vitamin D intake (14).

The effects of antiepileptic medicines on various exogenous and endogenous substances have been investigated. Some of antiepileptic drugs can cause vitamin D deficiency by influencing hepatic P450 system in the liver or disrupt 25-hydroxylation liver.

Some antiepileptic drugs may have an association with metabolism and bone mineral cycles due to the effects on bone cells (osteoblasts and osteoclasts) (15, 16). Our study showed that the prevalence of vitamin D deficiency and hypoparathyroidism was higher in patients. In a study that performed on 33 patients treated with antiepileptic drugs, 36% had insufficient levels of vitamin D deficiency and 58% had low level (17). Another study conducted on 38 children who received antiepileptic drugs and 44 healthy, 75% of patients had vitamin D deficiency, and 21% had insufficient vitamin D levels and the amounts of PTH were different between patients and controls (18). Another study conducted on effects of anti-epileptic drugs on bone metabolism. The levels of serum calcium and serum phosphate were normal in 93.76%, 93.3% of patients’ respectively .The ALP level was in the normal and higher than normal in 76.5% and 23.5% of patients respectively. The PTH levels were in the normal and higher than normal in 77.3% and 18.5% of patients. Increasing the level of alkaline phosphatase and PTH was significantly higher in patients than the control group (19). A similar study was conducted with the aims of assessing the effects of anticonvulsants on vitamin D metabolism in children with epilepsy, which 89 children with epilepsy and taking antiepileptic drugs for at least six months without underlying disease were included. About 42% of patients had vitamin D deficiency. Serum calcium and phosphate levels were in the normal range. Vitamin D levels were not statistically significant in some factors such as age group, sex, type of medicines for the treatment, therapy or Monotherapy Bridge and resistance to treatment (20). Similar to our results, in another study, the levels of vitamin D did not show differences accordance with the variables of age, sex, type of medication for the treatment, therapy or Monotherapy Bridge and of 111 patients, 22% were in the defence level of vitamin D and 41% had insufficient vitamin D (21). The results of this study were similar to our results.

**In Conclusion, **the frequency of hyperparathyroidy and vitamin D deficiency was higher in epileptic children. Supplemental vitamin-D administration in such patients may be helpful. Measuring calcium and vitamin D levels are essential in epileptic patients, especially when remembering that they are at higher risk of falling and bone fractures.

## Author`s Contribution

Nakhaee, Khajeh carried out the experiment and design. Nakhaee supervised the project and Khajeh supported. Hosseini performed data collection and Teimouri involved with data analysis and writing the primary version of manuscript.
